# The association between hyperuricemia and atrial fibrillation recurrence after catheter ablation

**DOI:** 10.1002/joa3.13030

**Published:** 2024-03-26

**Authors:** Hirotsuna Oseto, Seigo Yamashita, Michifumi Tokuda, Hidenori Sato, Satoko Shiomi, Ryutaro Sakurai, Masaaki Yokoyama, Kenichi Tokutake, Mika Katoh, Satoru Miyanaga, Michihiro Yoshimura, Teiichi Yamane

**Affiliations:** ^1^ Division of Cardiology, Department of Internal Medicine The Jikei University School of Medicine Tokyo Japan

**Keywords:** atrial fibrillation, catheter ablation, hyperuricemia, uric acid

## Abstract

**Background:**

Hyperuricemia (HU) has been reported to be associated with a high incidence of atrial fibrillation (AF). However, the relationship between HUA and recurrent AF after catheter ablation (CA) is unclear.

**Methods:**

Four hundred consecutive AF patients (paroxysmal/persistent AF [PAF/PsAF]: 200/200) who underwent the initial CA were retrospectively enrolled. HU was defined as serum uric acid (SUA) level >7.0 mg/dL. We measured SUA levels 1 day before (pre‐CA) and 1 month after CA (post‐CA). A second‐generation 28 mm cryoballoon was used for pulmonary vein isolation (PVI) for PAF, while PVI plus linear ablation (roof and mitral isthmus lines) by radiofrequency catheter was conducted for PsAF.

**Results:**

During 57 ± 24 months of follow‐up, AF recurred in 16% and 42% in PAF and PsAF patients (*p* < .0001). Pre‐CA SUA level in PsAF was significantly higher than that in PAF (6.5 ± 1.3 vs. 5.8 ± 1.3 mg/dL, *p* < .001). SUA level was significantly decreased after CA in both PAF and PsAF (5.8 ± 1.3 vs. 5.6 ± 1.3 mg/dL; *p* < .01 and 6.5 ± 1.3 vs. 6.1 ± 1.2 mg/dL; *p* < .0001, respectively). The association between pre−/post‐CA HU and recurrent AF was not identified in PAF, while the incidence of post‐CA HU was significantly higher in patients with recurrent AF than those without in PsAF (36% vs. 15%, *p* < .001). In multivariable analysis, longer AF duration and the presence of post‐CA HU were identified as independent predictors of AF recurrence in PsAF (OR:1.01, 95%CI:1.003–1.011, *p* = .0001 and OR:2.77, 95%CI:1.333–5.755, *p* = .007, respectively).

**Conclusions:**

SUA level was significantly higher in PsAF than PAF patients. The presence of post‐CA HU was strongly related to AF recurrence in PsAF patients.

## Graphical abstract

Baseline serum uric acid (SUA) level was significantly higher in PsAF patients than PAF patients. SUA level was significantly decreased at 1‐month after catheter ablation (CA) in both PAF and PsAF. The presence of hyperuricemia (HUA: >7 mg/dL) at 1‐month after CA was strongly associated with AF recurrence in PsAF, but not in PAF.

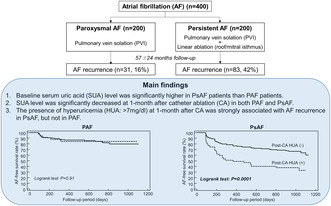

## 1 INTRODUCTION

Atrial fibrillation (AF) is widely known to be the most common arrhythmia in clinical practice. Multi‐risk factors for the development of AF have been reported.^1^, ^2^, ^3^, ^4^ Hyperuricemia (HU) has been also reported to be one of factors associating with development of AF^5^, ^6^ and related to chronic kidney disease, metabolic syndrome, left ventricular (LV) dysfunction, and all‐cause mortality.^7^, ^8^, ^9^, ^10^, ^11^

Regarding treatments of AF, catheter ablation (CA) is now a first‐line therapy and pulmonary vein isolation (PVI) is a gold standard method with a high success rate (70%–80%) in paroxysmal AF (PAF); however, ablation strategies for persistent AF (PsAF) are still under debate due to a modest success rate (50%–60%).^12^ Many predictors of the recurrence of AF after CA have been reported^13^; however, the prognostic significance of serum uric acid (SUA) levels with respect to the outcomes of AF ablation remain controversial.^14^, ^15^ The aim of this study was to assess the association between HU and the recurrence of AF after CA according to AF type.

## 2 METHODS

### 2.1 Study population and pre‐procedural evaluation

Among a total of 801 AF patients who underwent the initial CA between July 2014 and September 2017 in our institution, we enrolled the consecutive 200 PAF patients treated with cryoballoon ablation and 200 PsAF patients treated with PVI plus linear ablation, and who could be followed up at least 6 months and whose blood samples including SUA were obtained both pre‐ and postablation procedure were retrospectively enrolled. PAF was defined as AF lasting less than 7 days with or without antiarrhythmic drugs, while PsAF was defined as AF lasting over 7 days.^16^ The present study was approved by the ethics committee of The Jikei University School of Medicine for Human Research (29‐361(8977)), and informed consent was obtained with an opt‐out method in all patients.

### 2.2 Blood sample collection

Blood samples were collected and analyzed at pre‐CA (1 day before the procedure) and post‐CA (1 month after the procedure). The SUA concentration and other chemistry parameters were measured using standard laboratory procedures. HU was defined as SUA level >7.0 mg/dL in this study.^17^ Blood samples were collected at 3 and 6 months after the ablation procedure.

### 2.3 Ablation procedure

#### 2.3.1 Cryoballoon ablation for PAF

The detailed method was described in our previous study,^18^ in brief a 28‐mm second‐generation cryoballoon (ARC‐Adv‐CB, Arctic Front Advance; Medtronic, Inc, Minneapolis, MN) with a spiral mapping catheter (Achieve; Medtronic) was advanced to PV orifice. Applications with freezing time of 180–240 s were applied in each PV. If the PV potential remained after 2 cryoballoon applications, touch‐up ablation with an irrigated ablation catheter was added to achieve complete isolation.

#### 2.3.2 Radiofrequency ablation for PsAF

Radiofrequency (RF) guided‐PVI was performed as previously described elsewhere.^19^ In brief, individually segmental PVI was performed at the PV antrum by point‐by‐point application methods under guidance with a large‐size of 20‐polar circular catheter (20–25 mm Lasso, Biosense Webster, Inc., Diamond Bar, CA). In addition to PVI, the roof line and posterolateral mitral isthmus (MI) line were added in all cases.^20^, ^21^ RF energy was delivered for 30–60 s at each point using a 3.5 mm‐tip contact force sensing irrigated catheter (TactiCath™; Abbott or ThermoCool SmartTouch®/SF; Biosense Webster) with a power limit of 20–35 W and target CF of 10–30 g. The end point of PVI and linear ablation was the establishment of a bi‐directional conduction block between the LA and each PV and beyond each line.

### 2.4 Patient follow‐up

Twelve‐lead electrocardiograms (ECG) and 24‐h Holter ECG were recorded at 1, 3, 6 and 12 months and after CA and every 6 months thereafter. Recurrent AF was defined as the presence of AF lasting over 30 s after the blanking period (3 months). We encouraged patients to quit drinking alcohol before and after CA. Transthoracic echocardiography (TTE) was routinely performed just before and at 3 months after CA in all patients.

### 2.5 Statistical analysis

Continuous variables are expressed as the mean ± SD. Differences in continuous variables between the PAF group and PsAF group were analyzed by Student's *t*‐test or the Mann–Whitney *U*‐test. Differences in dichotomous variables were analyzed by the *χ* ^2^ test, except when values in any cell were <5, in which case Fisher exact test was applied. A linear regression analysis with the coefficient of determination (*R* ^2^) was used to assess the relationship between SUA and other clinical factors. Kaplan–Meier curves were used to analyze the rate of freedom from AF, and groups were compared using the log‐rank test. A multivariable analysis with Cox proportional‐hazards regression was used to analyze predictors of recurrent AF after the initial CA procedure. All statistical analyses were performed using the MedCalc software package, version 11.2 (MedCalc Software, Mariakerke, Belgium). *p‐*values of <.05 were considered to indicate statistical significance.

## 3 RESULTS

### 3.1 Patients' characteristics

As shown in Table 1, age, body mass index (BMI), LA diameter (LAD) and brain natriuretic peptide (BNP) value were significantly higher, and the LV ejection fraction (LVEF) was significantly lower in PsAF patients than PAF patients. Moreover, pre‐CA SUA and pre‐CA γ‐glutamyl transpeptidase (γGTP) levels in PsAF were significantly higher than those in PAF. Thus, the incidence of HU in PsAF was significantly higher than that in PAF (36% vs. 17%, *p* < .0001). Pre‐CA SUA level was mildly correlated with pre‐CA γGTP level (*R* ^2^ = 0.035, *p* < .01) and BMI (*R* ^2^ = 0.020, *p* = .045), but not with the levels of inflammatory markers, including WBC count and CRP level (*R* ^2^ = 0.008, *p* = .23 and *R* ^2^ = 0.004, *p* = .40, respectively). In this population, pre‐ and post‐CA SUA level was higher in male than in female (6.6 ± 1.3 vs. 5.9 ± 1.4 mg/dL, *p* < .05, 6.1 ± 1.2 vs. 5.5 ± 1.3 mg/dL, *p* < .05, respectively) and SUA‐lowering medications were prescribed in 39 (9.8%) and 40 (10%) patients before and after the procedure, respectively.

**TABLE 1:** Patients' characteristics.

	Total (*n* = 400)	PAF (*n* = 200)	PsAF *N* = 200	*p*‐value
Pre‐CA (1 day before CA)				
Age, years	58 ± 10	59 ± 11	57 ± 9	**<.01**
Sex, male	353 (88%)	171 (86%)	182 (91%)	.12
BMI, kg/m^2^	24.3 ± 3.3	23.5 ± 3.1	25.2 ± 3.2	**<.001**
History of AF, years	3.1 ± 3.7	3.1 ± 3.8	3.0 ± 3.7	.76
AF duration, months	–	–	22 ± 43	**–**
Hypertension, *n*	127 (64%)	52 (26%)	75 (38%)	**<.05**
Diabetes mellitus, *n*	31 (8%)	16 (8%)	15 (8%)	1.00
Heart failure, *n*	24 (6%)	1 (1%)	23 (12%)	**<.001**
CHADS_2_ score	0.6 ± 0.7	0.6 ± 0.8	0.6 ± 0.7	.42
LAD, mm	39.6 ± 6.3	36.3 ± 4.8	42.8 ± 5.8	**<.001**
LVEF, %	62.7 ± 6.9	64.7 ± 4.7	60.7 ± 8.1	**<.001**
eGFR, mL/min/1.73 m^2^	75.5 ± 12.8	76.6 ± 12.9	74.4 ± 12.7	.23
BNP, pg/mL	72.1 ± 80.4	47.7 ± 61.1	98.7 ± 90.1	**<.001**
WBC, ×1000/μL	5700 ± 1600	5800 ± 1700	5600 ± 1600	.43
CRP, mg/dL	0.11 ± 0.24	0.11 ± 0.30	0.11 ± 0.18	.94
Pre‐CA SUA, mg/dL	6.1 ± 1.3	5.8 ± 1.3	6.5 ± 1.3	**<.001**
Pre‐CA HU (>7 mg/dL), *n*	108 (27%)	33 (17%)	75 (36%)	**<.001**
Pre‐CA γGTP, mg/dL	54 ± 51	45 ± 41	63 ± 57	**<.001**
Use of SUA‐lowering medications, *n*	39 (10%)	15 (8%)	24 (12%)	.18
Post‐CA (1‐month after CA)				
Post‐CA SUA, mg/dL	5.8 ± 1.3	5.6 ± 1.3	6.1 ± 1.2	**<.001**
Post‐CA HU (>7 mg/dL), *n*	80 (20%)	33 (17%)	47 (24%)	.10
Post‐CA γGTP, mg/dL	52 ± 42	42 ± 32	60 ± 47	**<.001**
SUA reduction rate, %	4.3 ± 14	2.5 ± 15	6.1 ± 13	**<.01**
Use of SUA‐lowering medications, *n*	40 (10%)	15 (8%)	25 (13%)	.13

Abbreviations: AF, atrial fibrillation; BNP, brain natriuretic peptide; CA, catheter ablation; CRP, C‐reactive protein; eGFR, estimated glomerular filtration rate; HU, hyperuricemia; LAD, left atrial diameter; LVEF, left ventricular ejection fraction; PAF, paroxysmal AF; PsAF, persistent AF; SUA, serum uric acid; WBC, white blood cell; γGTP, γ‐glutamyl transpeptidase.

*p*‐values of <0.05 are bold values.

### 3.2 Ablation results

In PAF patients, all PVs were successfully isolated; 88% (707/800) of PVs were isolated by cryoballoon ablation alone with a mean of 5.0 ± 1.1 applications per patient. The remaining 93 PVs required additional touch‐up ablation by RF application. In PsAF patients, all PVs were successfully isolated, and roof and MI lines were completely established in 94% and 76%, respectively.

### 3.3 SUA levels before and after the initial CA procedure

**FIGURE 1:**
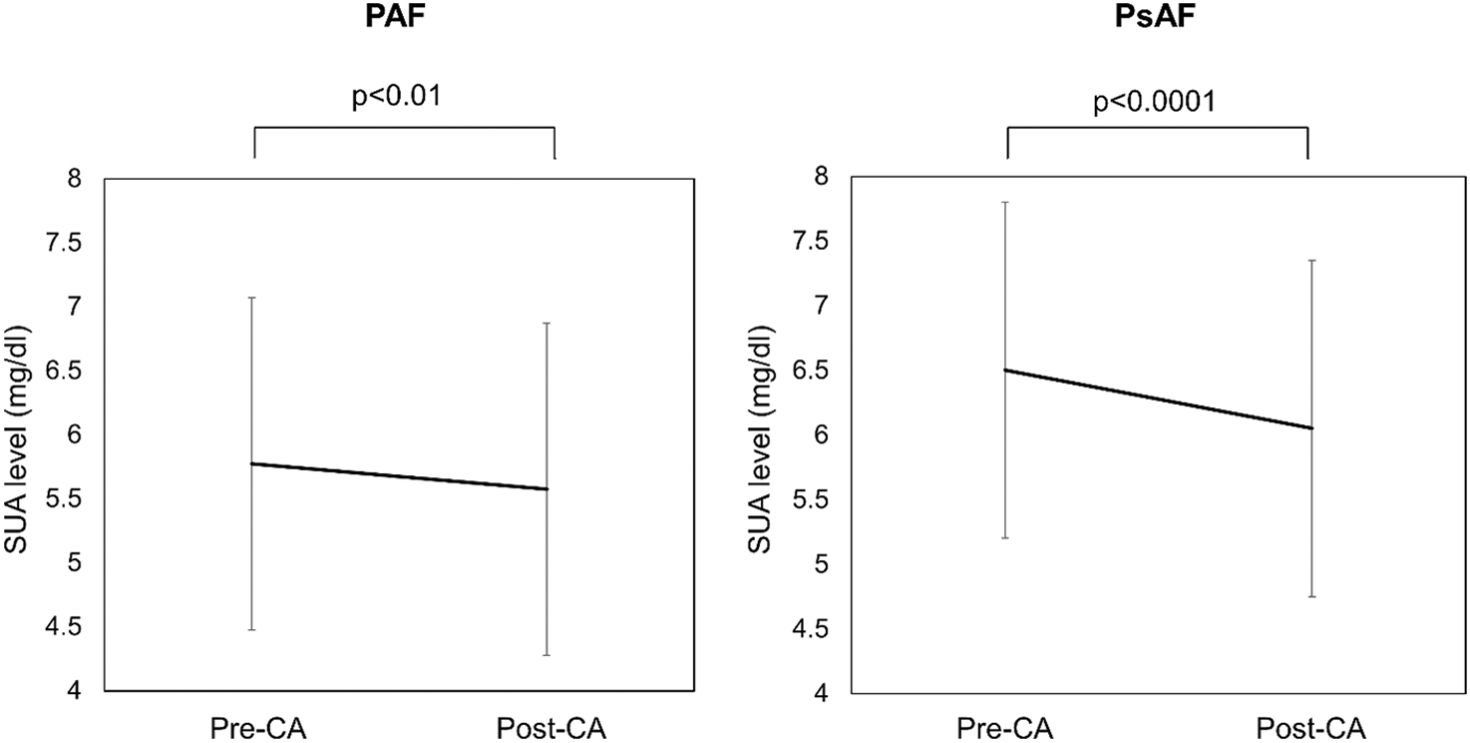
Pre‐ and Post‐CA SUA levels in PAF and PsAF patients. CA, catheter ablation; PAF, paroxysmal atrial fibrillation; PsAF, persistent AF; SUA, serum uric acid.

Figure 1 shows a comparison of pre‐ and post‐CA SUA levels in PAF and PsAF. In both groups, SUA level was significantly decreased after CA (5.8 ± 1.3 vs. 5.6 ± 1.3 mg/dL, *p* < .01 and 6.5 ± 1.3 vs. 6.1 ± 1.2 mg/dL, *p* < .0001). In addition, the incidence of HU was significantly decreased after CA in PsAF (36% vs. 24%, *p* < .001), but that was similar in PAF (17% vs. 17%, *p* = 1.00). The SUA reduction rate calculated as (pre‐post)‐CA SUA/pre‐CA SUA ×100 was also larger in PsAF compared with PAF (Table 1). On the other hand, pre‐ and post‐CA γGTP levels did not show any remarkable change in both PAF and PsAF (*p* = .07).

### 3.4 Predictors of AF recurrence after the initial CA procedure

**FIGURE 2:**
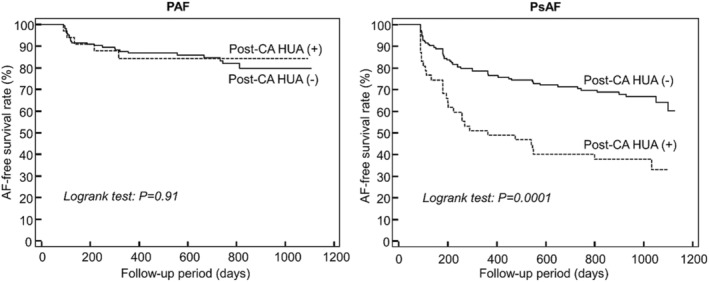
Kaplan–Meier curves for AF‐free survival rate in patients with and without post‐CA HU after the initial CA procedure in PAF and PsAF patients (HU: SUA level > 7.0 mg/dL). AF, atrial fibrillation; CA, catheter ablation; HU, hyperuricemia; PAF, paroxysmal AF; PsAF, persistent AF.

During 57 ± 24 months of follow‐up, AF‐free rate of PAF patients was significantly higher than that of PsAF patients (84% and 58%, log‐rank test: *p* < .0001). In PAF patients, BNP value was significantly higher in patients with AF recurrence than those without (*p* < .01). However, pre‐ and post‐CA SUA levels and the incidence of HU did not differ between the two groups. On the other hand, AF duration was significantly longer, and the incidence of post‐CA HU was significantly higher in patients with AF recurrence than those without in PsAF (35 ± 58 vs. 12 ± 25 months, *p* < .001 and 36% vs. 15%, *p* < .001, respectively, Table 2). In addition, post‐CA SUA level tended to be higher in AF recurrent group (*p* = .09), while SUA reduction rate after CA was not associated with AF recurrence (Table 2). In both PAF and PsAF, it is noteworthy that there was no significant difference in the acute ablation results between patients with and without AF recurrence (Tables S1 and S2). In multivariable analysis, a higher BNP value was the only independent predictor of AF recurrence in PAF (HR:1.01, 95%CI:1.002–1.008, *p* = .005), while a longer AF duration and incidence of post‐CA HU were independent predictors of AF recurrence in PsAF (HR:1.01, 95%CI:1.003–1.011, *p* = .0001 and HR:2.77, 95%CI:1.333–5.755, *p* = .007, respectively). A Kaplan–Meier curve clearly demonstrated a poor clinical outcome in patients with post‐CA HU in comparison to those without (log‐rank test: *p* = .0001) in PsAF. However, no difference was observed in PAF (log‐rank test: *p* = .91) (Figure 2). In addition, the similar relationship between HU and AF recurrence was still observed even in blood samples obtained at 3 and 6 months after CA. After 3 months of follow‐up, PsAF patients without AF recurrence demonstrated a significant decrease in LAD in comparison with those with AF recurrence (−7.2 ± 12.6 vs. −1.9 ± 4.5 mm, *p* = .003). Although we assessed the clinical outcome by using other cutoff values of post‐CA SUA (6.0 and 8.0 mg/dL), there was no significant difference between the PsAF patients with and without AF recurrence in both cutoff values (Figure S1).

**TABLE 2:** Clinical factors in patients with and without AF recurrence according to AF types.

	PAF (*n* = 200)			PsAF (*n* = 200)		
	AF rec (+)	AF rec (−)	*p*‐value	AF rec (+)	AF rec (−)	*p*‐value
	*n* = 31	*n* = 169		*n* = 83	*n* = 117	
Age, years	61 ± 10	59 ± 11	.50	58 ± 11	59 ± 9	.95
Sex, male	24 (77%)	137 (88%)	.32	76 (92%)	104 (89%)	.94
BMI, kg/m^2^	23.5 ± 3.1	23.6 ± 3.1	.94	26.5 ± 4.8	25.4 ± 3.8	.47
AF history, years	3.9 ± 4.0	2.9 ± 3.7	.18	4.0 ± 3.9	2.7 ± 3.2	**<.01**
AF duration, months	–	–	–	12 ± 25	35 ± 58	**<.001**
Hypertension, *n*	10 (32%)	42 (25%)	.52	28 (34%)	47 (40%)	.44
Diabetes mellitus, *n*	4 (13%)	12 (7%)	.46	7 (8%)	8 (7%)	.88
Heart failure, *n*	1 (3%)	0 (0%)	.34	7 (8%)	16 (14%)	.36
CHADS_2_ score	0.6 ± 0.8	0.6 ± 0.7	.85	0.8 ± 0.9	0.7 ± 0.8	.38
LAD, mm	36.7 ± 4.7	36.3 ± 4.9	.65	43.6 ± 5.0	42.6 ± 5.5	.26
LVEF, %	63.3 ± 5.1	64.9 ± 4.7	.11	63.6 ± 7.9	63.1 ± 8.4	.78
eGFR, mL/min/1.73 m^2^	76 ± 14	77 ± 13	.69	72 ± 12	73 ± 14	.56
BNP, pg/mL	75 ± 104	43 ± 48	**<.01**	112 ± 84	96 ± 127	.62
WBC, ×1000/μL	5.9 ± 2.6	5.8 ± 1.4	.87	5.5 ± 1.7	5.2 ± 1.8	.55
CRP, mg/dL	0.06 ± 0.04	0.12 ± 0.32	.34	0.11 ± 0.17	0.11 ± 0.18	.87
Pre‐CA SUA, mg/dL	5.8 ± 1.2	5.8 ± 1.3	.94	6.6 ± 1.4	6.4 ± 1.3	.28
Pre‐CA HU, *n*	3 (9%)	30 (18%)	.40	36 (43%)	39 (33%)	.19
Pre‐CA γGTP, mg/dL	43 ± 40	46 ± 42	.82	69 ± 72	58 ± 42	.19
Post‐CA SUA, mg/dL	5.5 ± 1.2	5.6 ± 1.3	.62	6.2 ± 1.3	5.9 ± 1.3	.09
Post‐CA HU, *n*	5 (16%)	28 (17%)	.84	30 (36%)	17 (15%)	**<.001**
Post‐CA γGTP, mg/dL	36 ± 28	43 ± 32	.33	66 ± 59	55 ± 35	.09
SUA reduction rate, %	3.9 ± 15	2.3 ± 15	.60	5.0 ± 13	6.9 ± 13	.30
Use of SUA‐lowering medications, *n*	4 (13%)	11 (7%)	.38	11 (13%)	13 (11%)	.81

Abbreviations: AF, atrial fibrillation; BNP, brain natriuretic peptide; CA, catheter ablation; CRP, C‐reactive protein; eGFR, estimated glomerular filtration rate; HU, hyperuricemia; LAD, left atrial diameter; LVEF, left ventricular ejection fraction; PAF, paroxysmal AF; PsAF, persistent AF; SUA, serum uric acid; WBC, white blood cell; γGTP, γ‐glutamyl transpeptidase.

*p*‐values of <0.05 are bold values.

## 4 DISCUSSION

The present study investigated the relationship between pre−/post‐CA SUA levels and AF recurrence after CA in PAF and PsAF patients. Our results have several important clinical implications: (1) SUA level differed according to the type of AF, (2) SUA level was significantly decreased at 1 month after CA in both PAF and PsAF, and (3) pre‐ and post‐CA SUA levels and the incidence of HU did not affect the clinical outcome after CA in PAF; however, the presence of post‐CA HU was strongly related to AF recurrence in PsAF. This is the first report to demonstrate a relationship between post‐CA HU and AF recurrence after CA, which suggests that excessive SUA product, even after CA, may be associated with the development of recurrent AF in PsAF patients.

### 4.1 HU and AF

Although many previous cross‐sectional, prospective cohort studies and meta‐analyses reported the association between HU and the incidence of AF,^5^, ^6^, ^22^ the precise mechanisms are not fully understood because of the presence of numerous confounding factors. Based on basic research, SUA is the end product of purine degradation catalyzed by xanthine oxidase (XO), which has been reported to induce endothelial dysfunction, oxidative stress,^23^ and systemic inflammatory responses.^24^ Thus, SUA level is thought to be a marker of tissue oxidative stress and inflammation, which leads to the generation of atrial remodeling as an AF substrate.^25^, ^26^ Letsas et al. clinically demonstrated that higher SUA levels in PsAF compared with PAF, which could be attributed to different underlying pathophysiological triggers or electrical remodeling in PAF and structural remodeling in PsAF.^27^ Moreover, Chao et al. previously presented a positive correlation between the LAD and SUA levels,^28^ and the enzymatic activity of XO in the LA and LA appendage in AF patients has been reported to be greater than that in controls.^29^ These previous reports suggested that the production of UA was increased in the chronic stage of AF. In our study, the LAD was larger in PsAF than PAF, and it is noteworthy that the SUA level was significantly higher in PsAF than PAF, while the levels of inflammatory markers (WBC, CRP) did not differ between PAF and PsAF. A potential explanation might be that—rather than inflammation—the SUA level more selectively reflected increased atrial oxidative stress.

### 4.2 HU and recurrent AF after CA

The several previous studies have shown that high pre‐SUA level is a predictor of AF recurrence after CA; however, conflicting results have also been reported.^14^, ^15^ Canpolat et al. prospectively showed that increased pre‐CA SUA levels were associated with a higher rate of recurrence of AF after cryoballoon ablation in PAF patients.^15^ Their results was not consistent with our results. The differences in the patient population including younger age (53.5 ± 11.2 years), high female ratio (47.4%), higher CHADS2 score (1.52 ± 0.81), and shorter follow‐up period (19.2 ± 6.1 months), might have been responsible for the conflicting results; furthermore, there was no mention about the risk management after CA. In contrast, the most recent meta‐analysis including four cohort studies could not prove the significant association between high pre‐CA SUA levels and the increased risk of recurrence of AF after CA.^30^ The designs of these studies were quite heterogenous regarding AF types (PAF or PsAF), duration of follow‐up and ablation strategy, thus it is difficult to draw a conclusion. The present study was the first report to assess pre‐CA and post‐CA SUA levels and demonstrated the relationship between post‐CA HU (>7 mg/dL) and recurrent AF after CA in PsAF patients but not PAF patients, which might suggest that activated XO due to advanced atrial remodeling—even after sinus restoration by CA—could be associated with post‐HU and the re‐initiation of AF. This result can be explained by the fact that recurrent AF is mainly related to PV reconnection in PAF, while the degree of LA remodeling is more important in PsAF.^31^ Based on previous research demonstrating a positive correlation between the LAD and SUA levels,^28^ irreversible atrial structural remodeling or remaining triggers after CA might promote sources of arrhythmia for the recurrence of AF. In fact, a previous study demonstrated that the presence of reverse remodeling in the LA volume after CA was one of the predictors of a successful outcome.^32^ Our study also demonstrated less reverse remodeling of the LA in patients with recurrent AF in comparison with those without 3 months after CA. The other possible explanation regarding post‐CA HU as a predictor of the recurrence of AF might be related to strict habitual management after CA. Pathak et al. elegantly showed the effectiveness of aggressive risk factor management after CA.^33^ In our study, we strongly recommended that patients quit alcohol, which may reduce SUA levels and lead to higher success rate in patients who could improve their habitual risk factors after CA. In fact, the previous study demonstrated that the frequency and degree of alcohol consumption was a risk factor for HU,^34^ therefore both SR maintenance and reduction in alcohol consumption after CA were assumed to be causes of SUA reduction in the present study. However, risk management was not assessed during follow‐up in this study, thus further studies will be needed to clarify the relationship between clinical outcomes and risk management, including post‐CA SUA levels after CA.

## 5 STUDY LIMITATIONS

The present study had some limitations. First, this was a retrospective observational study with a relatively small sample size and a heterogenous population (only 12% of the patients were female) in a single center. Further investigation will be needed to clarify gender differences. Second, we may have underestimated the incidence of recurrent AF due to a lack of symptoms. Third, we did not assess risk management regarding alcohol consumption before and after the procedure, therefore the relationship between poor management of habitual risk factors and HU after the procedure was unclear. However, HU after the procedure related to AF recurrence in patients with not PAF but PsAF under the same risk management. This result suggested that HU might be affected by not only habitual risk factors but also a product of atrial oxidative stress and inflammation. Fourth, the use of different ablation modalities for PV isolation between PAF and PsAF may affect the clinical outcome, but the ablation strategy was same in each group, therefore the ablation strategy bias is considered to be small when we compared the outcome in patients with and without HU. Last, it is still unclear whether HU can be therapeutic target to cure AF after CA. A further prospective investigation regarding the effectiveness of SUA‐lowering medications after CA will be required.

## 6 CONCLUSIONS

The pre‐CA SUA level in PsAF was significantly higher than that in PAF. Although pre‐ and post‐CA SUA levels were not associated with AF recurrence after CA in PAF patients, the presence of post‐CA HU strongly related to AF recurrence in PsAF patients.

## CONFLICT OF INTEREST STATEMENT

There is no COI to disclose directly related to this study.

## Supporting information


Supplemental Figure 1.
