# Supplementary material for: The association between hyperuricemia and atrial fibrillation recurrence after catheter ablation
Source: J Arrhythm. 2024 Mar 26;40(3):520–6. doi: 10.1002/joa3.13030 (PMC11199822; doi:10.1002/joa3.13030)
Supplement: Supplementary file 1 — Supplemental Figure 1. [file JOA3-40-520-s001.docx]

**SUPPLRMENTAL TABLES**

**Supplemental Table 1.** Ablation results in PAF patients with and without AF recurrence.

|  | AF recurrence (+)  n=31 | AF recurrence (-)  n=169 | P-value |
| --- | --- | --- | --- |
| Successful PVI, % | 100% | 100% | 1.0 |
| Total N of application, n | 5.2±1.0 | 5.0±1.1 | 0.29 |
| Touch up ablation (/pts) | 16 (52%) | 65 (38%) | 0.24 |
| Touch up ablation (/PVs) | 19 (15%) | 74 (11%) | 0.21 |
| LSPV | 3 (10%) | 9 (5%) | 0.60 |
| LIPV | 5 (16%) | 14 (8%) | 0.30 |
| RSPV | 2 (6%) | 6 (4%) | 0.80 |
| RIPV | 9 (29%) | 45 (27%) | 0.95 |
| Non-PV foci | 6 (19%) | 25 (15%) | 0.71 |
| SVC isolation | 5 (16%) | 21 (12%) | 0.78 |
| MI linear ablation | 1 (3%) | 2 (1%) | 0.96 |
| Dormant conduction (/pts) | 9 (7%) | 28 (4%) | 0.20 |

Abbreviations: AF, atrial fibrillation; LIPV, left inferior PV; LSPV, left superior PV; MI, mitral isthmus; N, number; PV, pulmonary vein; PVI, PV isolation; RIPV, right inferior PV; RSPV, right superior PV; SVC, superior vena cava.

**Supplemental Table 2.** Ablation results in PsAF patients with and without AF recurrence.

|  | AF recurrence (+)  n=83 | AF recurrence (-)  n=117 | P-value |
| --- | --- | --- | --- |
| Successful PVI, % | 100% | 100% | 1.0 |
| Non-PV foci, n | 22 (27%) | 26 (22%) | 0.60 |
| SVC isolation, n | 5 (6%) | 13 (11%) | 0.32 |
| Successful Roof line, n | 78 (94%) | 110 (94%) | 0.77 |
| Successful MI line, n | 61 (73%) | 91 (78%) | 0.60 |
| RF applications within CS | 47 (57%) | 72 (62%) | 0.58 |
| AT induction during procedure, n | 11 (13%) | 9 (8%) | 0.29 |

Abbreviations: AF, atrial fibrillation; AT, atrial tachycardia; MI, mitral isthmus; PV, pulmonary vein; PVI, PV isolation; RF, radiofrequency; SVC, superior vena cava.

**Supplemental Figure.** Kaplan-Meier curves for AF-free survival rate after the initial CA procedure in PAF and PsAF patients according to the different cut off value of SUA level (6 mg/dl and 8 mg/dl, respectively).


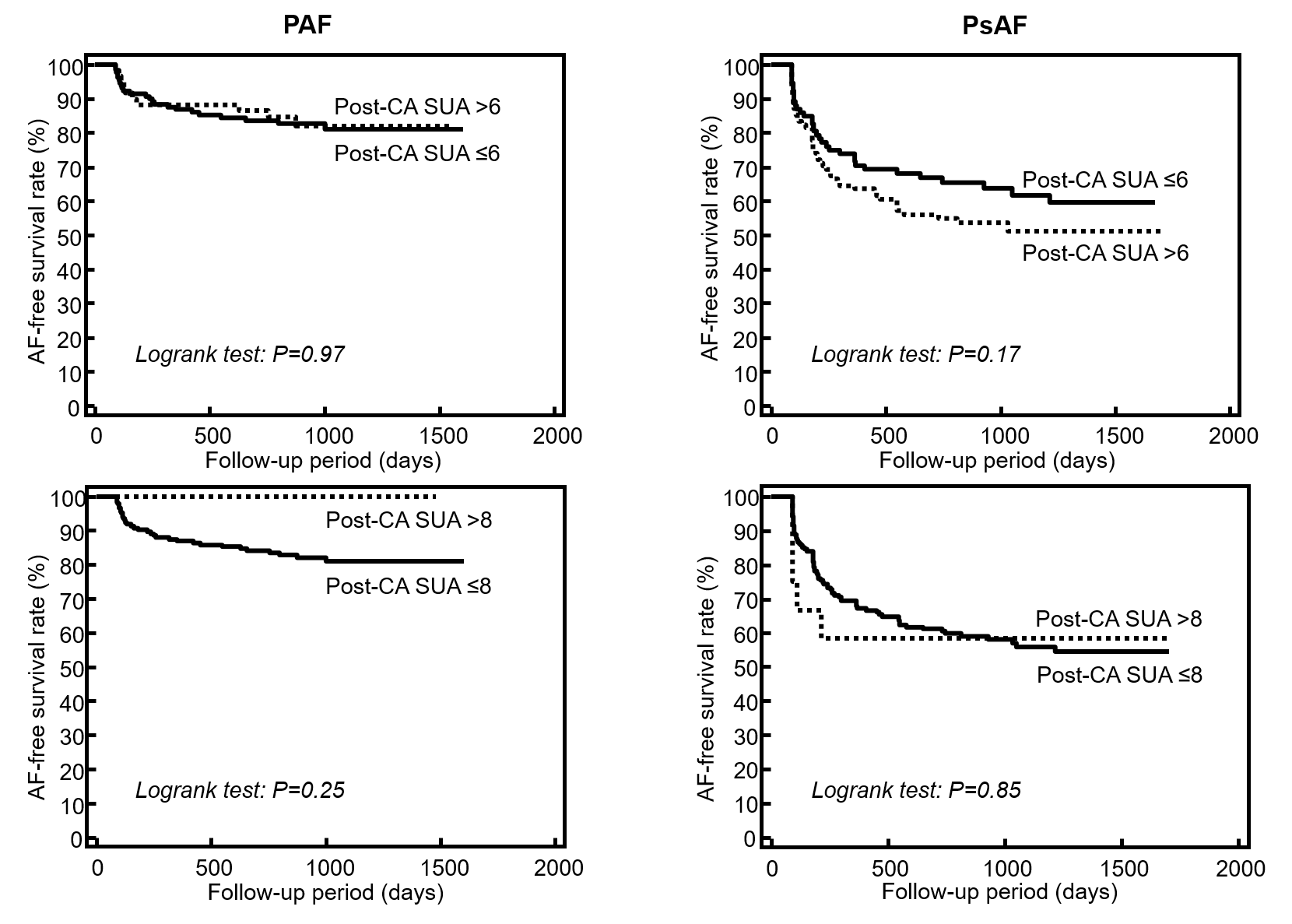


Abbreviations: AF, atrial fibrillation; CA, catheter ablation; PAF, paroxysmal AF; PsAF, persistent AF; SUA, serum uric acid.
